# ASIP Performance Enhancement by Hazard Control through Scoreboard

**DOI:** 10.3390/mi15111287

**Published:** 2024-10-23

**Authors:** Xinbing Zhou, Yi Man, Peng Hao, Wei Chen, Bo Yang, Baoguo Ding, Dake Liu

**Affiliations:** 1 School of Information and Communication Engineering, Hainan University, Haikou 570228, China; xinbingzhou@hainanu.edu.cn (X.Z.); weichen@hainanu.edu.cn (W.C.); 2 School of Electronic Engineering, Beijing University of Posts and Telecommunications, Beijing 100876, China; 3 School of Computer Science, Northwestern Polytechnical University, Xi’an 710072, China; peng.hao@mail.nwpu.edu.cn; 4 School of Electronic and Information Engineering, South China University of Technology, Guangzhou 510641, China; yangb@comba.com.cn (B.Y.); 202211082192@mail.scut.edu.cn (B.D.); 5 Ultichip Communication Technology, Beijing 100088, China; dake@hainanu.edu.cn

**Keywords:** ASIP design, data hazard, RAW/WAW, scoreboard, performance enhancement, digital signal processor, Domain specific architecture

## Abstract

The application-specific instruction set processor (ASIP) has been gradually accepted in AI, communication, media, game and industry control. The digital signal processor (DSP) is a typical ASIP, whose benefits include high performance in specific domains, low power consumption, high flexibility and low silicon consumption. One of the challenges for DSP design is to handle problems induced by datapath acceleration. The datapath acceleration (instruction fusion, black box instructions) induces control complexities. To most efficiently utilize hardware, control challenges can be summarized as RAW (Read-After-Write) handling, hardware hazard handling, and WAW (Write-After-Write) handling. Both an advanced compiler and hardware hazard handler can be used as solutions. In this paper, we introduced both solutions and exposed the benefits from the hardware solution. The benefits include utilizing low silicon to achieve higher performance and program memory reduction on chip. In summary, our solution only uses 0.91% extra silicon area yet achieves 32.75% performance improvement. So, the overall performance-to-cost ratio could be evaluated as 32%.

## 1. Introduction

According to John Hennessy’s analysis [1], over the course of more than 50 years’ development, the mainstream future of computing lies in heterogeneous architecture composed by domain-specific processors. These processors are characterized by instruction level acceleration in application domains, custom data types, the use of TCM (Tight Coupled Memory) to accelerate data retrieval, and often programming via DSL (Domain-Specific Language). DSP is a prevalent type of ASIP. Although DSPs have a more limited application range compared to general-purpose CPUs, they offer significant advantages such as lower silicon costs, reduced power consumption, and enhanced performance particularly in embedded systems and mobile devices [2]. In contrast to ASIC circuits, DSPs provide similar performance while maintaining greater flexibility, leading to broader adaptability and longer market viability [3].

A system designed for digital signal processing is referred to as a DSP system, which is widely employed across various fields, including AI, communication, media, gaming, and industrial control [4]. Parallel processing is the primary method for enhancing processor performance. Modern general-purpose processors often implement superscalar architectures to achieve instruction-level parallelism (ILP), while DSPs typically adopt very long instruction word (VLIW) architectures [5] to hide control and data access overheads. Data parallelism is achieved through SIMD (Single Instruction Multiple Data) or vector methodologies.

Application-oriented vector processors include general-purpose architectures such as AVX512 [6], ARM SVE [7], and RSIC-V RVV [8] as well as application-oriented DSP architectures like TI C66 [9], Qualcomm Hexagon [10], CEVA XC12 [11], XC16 [12], XC22 [13], and Tesla Dojo [14]. To facilitate compilation and user programming, most vector DSPs employ an RISC vector instruction architecture, where each instruction specifies a relatively limited function, which constrains the performance of DSPs. Based on application requirements and ASIP concepts [4], some DSPs combine multiple sequential instructions that are frequently used in single composite instruction while merging the RISC datapath into a multiple pipelined datapath [15]. This approach significantly enhances the performance of SIMD DSPs by accelerating frequently utilized functionalities. Consequently, the instruction set of ASIP DSPs differs from that of general-purpose processors. In certain fields, they often feature complex instruction set designs. For instance, in wireless communications, data are represented in IQ (In-phase, Quadrature) format, which is a complex data representation [16]. Common instructions for IQ data include complex addition, subtraction, and multiplication as well as multistep fused instructions, such as summation, minimum, and maximum calculations. The typical features of these instructions can be identified not only data parallel but also instruction fusion, the integration of multi-operations into one instruction, such as incorporating post-processing tasks like saturation, scaling, and rounding. Instruction fusion results in longer instruction pipelines. Extended pipelines can introduce data hazards, such as RAW (Read-After-Write), WAW (Write-After-Write) dependencies and hardware hazards. If data hazards are not effectively addressed, they can lead to significant performance degradation and even risks of computational errors.

To address the challenges, different solutions are provided, such as superscalar architecture and advanced compiler scheduling. However, the superscalar hardware overhead is high and the advanced compiler scheduling cannot handle dynamic dependencies. In this paper, we develop a low hardware cost solution, which is a hardware correlation management to maximize the utilization of the multiple pipelined datapath. It significantly enhanced processor efficiency (IPCs, Instructions Per Cycle). This constitutes the core contribution of this work.

The simplest solution to address data hazards is pipeline stalling, which is straightforward in design but significantly reduces IPC and overall processor performance. An alternative approach is to utilize compiler-based instruction scheduling [17]. For long pipeline instructions and their subsequent correlated instructions, unrelated instructions can be inserted in between or no-operation (NOP) instructions can be introduced, although this increases the code size. Another method is the Tomasulo algorithm, invented by Robert Tomasulo in 1967, which supports out-of-order (OoO) execution. This algorithm tracks when operands become available to minimize RAW hazards and incorporates register renaming in hardware to reduce WAW and WAR hazards [18]. However, OoO execution requires a reorder buffer (ROB) to ensure that instructions are written back in the correct order, resulting in substantial overhead [19]. While ROBs, along with support for ordered commit and branch prediction, enable modern processors to handle complex programming demands, they increase significantly hardware overheads, which is unacceptable for low-power DSPs.

Although ASIP enhances processing performance, it introduces challenges between performance and reliability. Recently, there have been articles addressing such challenges [20,21,22], yet the residual issues remain unresolved with low-cost hardware solutions. These unresolved issues include how to tackle RAW, WAW, and hardware hazard problems for long pipeline executions with low-cost hardware. This paper proposes a method to address these challenges without relying on expensive OoO hardware. Specifically, it introduces a scoreboard module for data hazard avoidance suitable for ASIP DSPs. We thus significantly enhance the performance of long pipeline ASIP DSP effectively and maintain extremely low overhead. In the target processor, this approach incurs only a 0.97% increase in silicon cost while delivering a 32.75% performance boost.

The structure of this paper is organized as follows: Section 2 presents the background and motivation; Section 3 details our proposed algorithm and implementation scheme; Section 4 discusses the implementation and analyzes the results in comparison to existing methods; and last, Section 5 concludes the paper with a summary.

## 2. Background and Motivation

### 2.1. Target Processor

**Figure 1:**
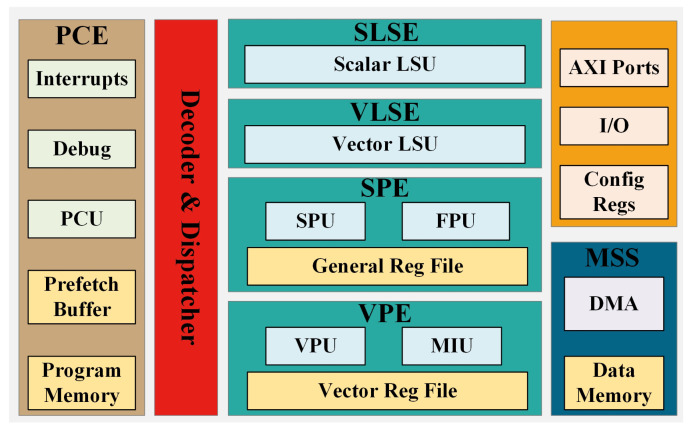
Sayram top level architecture diagram.

Sayram is a VLIW vector DSP designed by ULTICHIP, specifically targeting the wireless communication domain [23]. This DSP extends the RISC-V instruction set [24] with custom vector ALU (Arithmetic Logical Unit) instructions tailored for wireless communications, supporting complex arithmetic and specialized acceleration operations. Its top-level architecture is shown in Figure 1. The processor employs a Harvard architecture with separate data and program memory. The complexity of analyzing data hazards in VLIW DSPs can be measured in terms of both spatial complexity and time complexity.

#### 2.1.1. Spatial Complexity

Sayram implements instruction parallelism through a VLIW architecture, featuring four slots: SPE (Scalar Processing Engine), VPE (Vector Processing Engine), SLSE (Scalar Load Store Engine), and VLSE (Vector Load Store Engine), allowing for the simultaneous execution of four instructions. SLSE is dedicated to scalar storage, while VLSE handles vector memory access. SLSE comprises two components: SPU (Scalar Processing Unit) and FPU (Float Processing Unit), both compatible with the RISC-V instruction set, supporting RV32I, RV32M, and RV32F instructions [24]. The VPE includes a VPU (Vector Processing Unit) and an MIU (MICRO Instruction Unit). The VPU is for general vector operations, enhancing datapath acceleration through instruction fusion, and the MIU supports black box instructions tailored for wireless communication processing, encompassing nonlinear operations, modulation, and demodulation.

**Figure 2:**
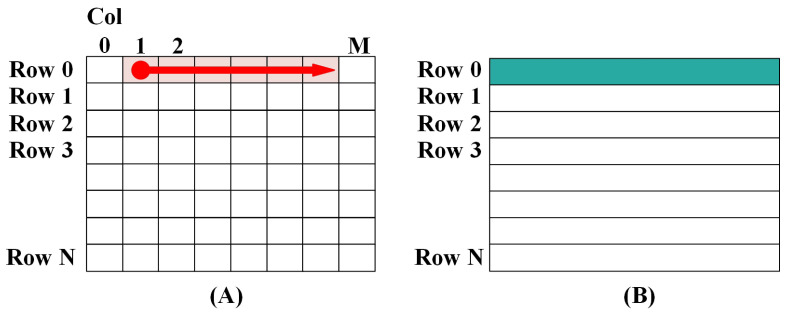
Vector register versus SIMD register. (**A**) shows vector register files mentioned in this project and the red arrow shows the start-point and length for the accessed vector; (**B**) is a common SIMD register file. N represents the total amount of rows, while M represents the column number.

In modern advanced processors, memory access and branching typically consume the most time [18]. To reduce memory access frequency and enhance processor performance, Sayram employs a specialized vector registers. Sayram adopts a true vector design approach, which differs significantly from traditional SIMD architectures, such as Intel AVX512 [6], ARM NEON [25], and RISC-V RVV [26], particularly in terms of register access methods. Figure 2 illustrates the distinction between the vector register file of Sayram and the traditional SIMD register file in terms of register read and write operations. In conventional SIMD architectures, registers read a long vector register at once, which is then decomposed into multiple units for parallel computation by the execution units. For example, Intel’s AVX512 instruction set reads a fixed 512-bit data block at a time, supporting computations with data types of 16-bit, 32-bit, and 64-bit. In contrast, Sayram enables true vector reads, where a vector is defined by a starting point and a length. Sayram employs the concepts of cell and unit accesses: a cell represents the data type, supporting 16 bit/32 bit/64 bit real data and complex data, while a unit indicates the minimum data width of 16 bits, serving as the smallest access unit for the vector register file.

The balance of vector register file design involves the register size, register file utility ratio, and the silicon cost (number of registers and the binary code length), and it is constrained by applications. Sayram’s vector register design methodology enables higher utilization ability, accommodating more data. However, this elaborate register access approach complicates the handling of data validity. For instance, the VPE features two read ports and one write port, while the VLSE has one read port and one write port with each port capable of handling data widths ranging from 16 to 512 bits. The complexity of the register read and write mechanisms exacerbates the occurrence of data hazards.

#### 2.1.2. Time Complexity

**Figure 3:**
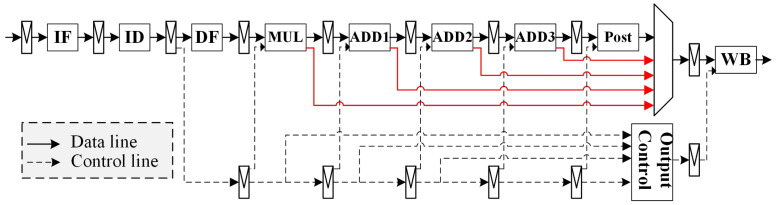
VPU pipeline with multiple pipeline endpoints emphasized by red lines. The red arrows show the different endpoints for ALU.

Figure 3 illustrates the pipelined architecture of the VPU. To enhance the efficiency of the DSP, Sayram combines multiple frequently used sequential instructions into a single composite instruction while merging the RISC datapath into a multiple pipelined datapath. The ALU features a multi-stage pipeline with each stage capable of serving as a pipeline endpoint write-back. This design provides a multitude of combinations, resulting in a rich functionality that enhances the utilization of the ALU and enables instructions to achieve higher IPC.

However, this approach involves trade-offs. The varying write-back times introduce complexity in determining RAW and WAW hazards and create issues with the simultaneous write-backs of consecutive instructions, leading to data hazards.

The flexibility in reading and writing vector registers, along with multiple write ports, increases spatial complexity, which can be represented as $O(n^{3})$. Additionally, from the perspective of the target DSP, the time complexity of data hazards in ASIP vector DSPs arises from differing write-back times due to varying pipeline lengths, which can be expressed as O(n).

### 2.2. Data Hazard Description

RAW refers to the situation where the execution of a later instruction depends on the write-back of an earlier instruction; WAW occurs when a later instruction writes to the same register as an earlier instruction. Hardware hazards arise when a later instruction and an earlier instruction simultaneously access the same hardware resource. These are three critical issues that ASIP DSPs need to address. To avoid RAW data hazards, it is essential to ensure that later instructions can read the write-back results of earlier instructions. To prevent WAW hazards, when both later and earlier instructions write back to the same target, the earlier instruction must complete first. Additionally, a common data hazard known as WAR (Write-After-Read) is not applicable in this context due to the in-order instruction issue. Thus, this paper will not discuss WAR further.

To illustrate RAW, WAW, and hardware hazards, we take the Sayram vector instruction set as an example. VLH is the vector load, which has four execution stages and one write-back (WB) stage; VADDH is a 16-bit vector addition operation with two execution stages and one WB stage; VSLLIH (16-bit vector logical left shift by immediate) has one execution stage and one WB stage.

Below are examples of RAW, WAW, and hardware hazards:

RAW: The third instruction’s reading depends on the write-back of the first instruction, meaning the third instruction must wait until the first instruction’s write-back is valid. The example instruction sequence is shown in Listing 1.

**Listing 1.** RAW example.1vlh vx3.0,x0,4,32 ; Read 512 bits from address x0 + 0 and write 32 shorts to vx3.0.2vsllih vx5.0,vx6.0,4,32 ; Perform a left shift of 4 bits on the 32 short data starting from vx6.0.3vsllih vx4.0,vx3.0,4,32 ; Perform a left shift of 4 bits on the 32 short data starting from vx3.0.

WAW: The write-back of the second instruction occurs in the same register location as that of the first instruction, meaning the second instruction should write back after the first instruction has completed. The example code for WAW is shown in Listing 2.

**Listing 2.** WAW example.1vlh vx3.0,x0,4,32 ; Read 512 bits from address x0 + 0 and write 32 shorts to vx3.0 - vx3.31.2vsllih vx3.0,vx4.0,4,32 ; Perform a left shift of 4 bits on the 32 short data starting from vx4.0 and write the result to vx3.0.

Hardware Hazard: Both the first and second instructions attempt to write back at the same time, resulting in a hardware conflict. The example code for hardware hazard is shown in Listing 3.

**Listing 3.** Hardware hazard example.1vaddh vx1.0,vx4.0,vx3.0,32 ; Perform a addition of 32 short data from vx4 and vx3 and write the result to vx6.2vsllih vx3.0,vx5.0,4,32 ; Perform a left shift of 4 bits on the 32 short data starting from vx5.0 and write to vx6.0.

**Figure 4:**
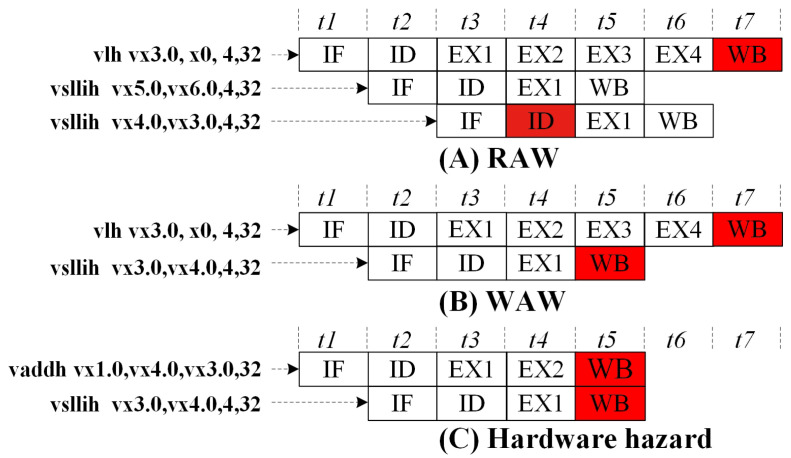
Data hazard description for (**A**) RAW, (**B**) WAW and (**C**) hardware hazard. The red squares represent where conflicts happen. IF for instruction fetch; ID for instruction decode; EX for execution and WB for write-back.

Figure 4 illustrates the execution process of the three instruction sequences with colored flows indicating where conflicts occur.

**Figure 5:**
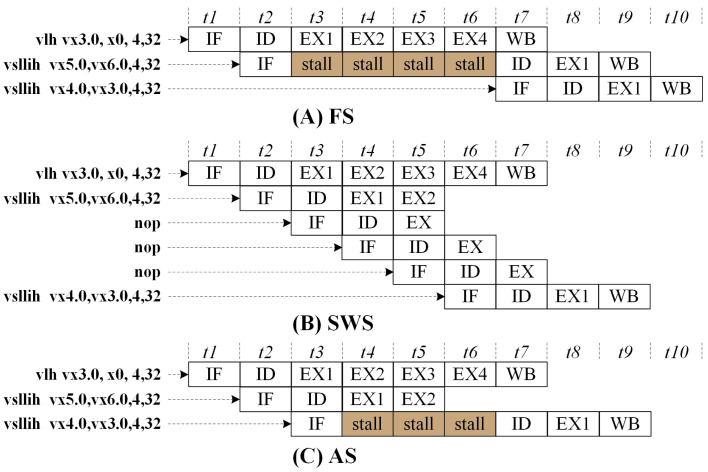
Processor running status for (**A**) FS, (**B**) SWS, and (**C**) AS architecture.

To address the aforementioned issues, superscalar processors employ register renaming and reservation techniques [19]. In contrast, DSPs typically utilize a complete stall hardware strategy, where the pipelined execution is blocked for instructions until the execution is completed, allowing subsequent instructions to issue later. This approach is logically straightforward and incurs few hardware overhead, which is referred to as full stall (FS). Alternatively, software-managed instruction scheduling can be employed to mitigate hazards, relying on advanced compilers to arrange instruction order and prevent data hazards, which is termed software schedule (SWS). The design concept presented in this paper introduces a scoreboard component that analyzes data hazards for predictable instruction behaviors and automatically stalls the pipeline, which is referred to as an auto-stall (AS). Figure 5 describes the processor’s different behaviors under these three strategies in the context of RAW as illustrated in Figure 4A.

Analyzing the three scenarios illustrated in Figure 5, the IPC for FS is 0.3, while for AS and SWS, the IPC is 0.33, representing a 20% improvement. In addition, SWS requires the addition of three extra NOPs to handle data hazards compared to FS; thus, SWS increased the instruction overhead by 100%.

### 2.3. Related Works

In industry, general-purpose processors often utilize OoO execution to address SIMD data hazards. Intel’s Alder Lake CPU employs an OoO architecture to implement AVX instructions [27], which could effectively handle data hazard issues. Due to commercial confidentiality, we are unable to access the specific implementation details of CEVA and Tensilica DSP IP. TI C66 [9] and Hexagon HVX [28] are typical SIMD DSPs with an open source instruction set. TI C66 uses a cross-path stall approach, which detects data correlation when an instruction attempts to read a register via a cross-path (also called forwarding path) that was updated in the previous cycle [9]. This design utilizes the cross-path to address the RAW issues between producer and consumer instructions based on each read and write port. However, it does not resolve the WAW problem, which would be later addressed by software. There is interlock stall logic for Hexagon HVX when an early source register was produced in the previous vector packet, but they prefer to rely on the software to schedule an intervening packet [28].

Let us take a glance at the literature. Ref. [20] explores the data hazard resolution in a simple five-stage pipeline RISC-V processor, utilizing a single-bit scoreboard to verify the validity of write-backs. This approach is suitable for short pipeline DSPs to tackle RAW and WAW hazards, but it is inefficient for detecting WAW in long pipelines. SiMT-DSP [21] presents a SIMT architecture DSP, where each sub-core employs a CISC design that utilizes full stall (FS) logic in the data pipeline to await valid data from memory. Ref. [22] designs a vector DSP with data hazard detection, featuring an equally long pipeline architecture, allowing vector register access by row or column. This design incorporates vector register data hazard detection during the instruction issue phase, evaluating hazards on a coarse-grained basis that is either row or column-wise. However, it lacks fine-grained vector register data hazard detection and exhibits low efficiency in WAW detection. None of these methods effectively address the data hazard issue in long pipeline DSPs.

Finally, we briefly introduce instruction scheduling. List scheduling is a general scheduling methodology. However, current DSP compilers are beginning to adopt heuristic algorithms to maximize parallel instruction execution and enhance processor efficiency. Ref. [29] describes an instruction-level scheduling heuristic, a modulo-based, register-sensitive algorithm that computes an efficient sequence of VLIW instructions for the DSP, maximizing parallelism while minimizing clock cycles and register utilization. Ref. [30] discusses a reinforcement learning (RL)-based model for instruction scheduling, incorporating processor features such as forwarding, resource utilization, and action space management. Additionally, ref. [31] introduces a unified instruction scheduling heuristic approach grounded in Worst-Case Execution Time (WCET) awareness, including dynamic register pressure control and basic block prioritization for integrated instruction scheduling.

## 3. The Proposed Architecture

To address the challenges mentioned above, this paper presents a scoreboard component aimed at enhancing DSP performance, allowing the processor to execute instructions in original order while automatically controlling instruction issuance based on data dependencies. This approach ensures that RAW, WAW, and hardware hazards do not occur. This architecture achieves significant performance improvements with minimal overhead.

### 3.1. Algorithm

Referring to concepts in compiler principle [17], data dependencies can be described for RAW and WAW as follows. Assume there are two statements, L1 and L2, where L1 executes before L2. Based on different access methods, data dependencies can be categorized into two types:

1. Flow Dependency: If there exists an executable path from L1 to L2, and L1 produces one or more outputs used as inputs by L2, then L2 has a flow dependency on L1, which raises an RAW hazard.
2. Output Dependency: If L1 and L2 write to the same output variable, there exists an output dependency between the two statements, rising a WAW hazard.

To resolve dependency issues, we must analyze the dependency relationships of each instruction with its predecessors. We can assess data conflicts based on register read and write times. Let $T_{w1}$ represent the write-back time of the preceding instruction, $T_{w2}$ the write-back time of the subsequent instruction, and $T_{r2}$ the subsequent instruction’s read time. To avoid RAW and WAW conflicts, the processor need to ensure Equation (1).

(1) $$T_{w1}<T_{r2}\;\cap \;T_{w1}<T_{w2}\;=\;1$$

Modern DSP designs often incorporate forwarding logic to bypass data from the WB port to the register read ports to save instruction cycles [22]. Therefore, the conditions for conflict avoidance change to Equation (2).

(2) $$T_{w1}\leq T_{r2}\;\cap \;T_{w1}<T_{w2}\;=\;1$$

To simplify the model, this project adopt the concept of relative time. Whenever an instruction is issued that requires writing to a register, the scoreboard need a status register to hold the value of $T_{w1}$ as the datapath pipeline depth of the instruction for that register. Each clock cycle, this value is decremented until it reaches zero. If subsequent instructions read or write to that register, they must evaluate Equation (2) to determine if they can issue. If not, a blocking signal is set; if they can issue and write back to the register, $T_{w}2$ is updated in the status register. Thus, we dynamically establish a dependency relationship table.

Regarding hardware hazards in Figure 4C, we need to manage potential write port conflicts. A dynamic occupancy table is need to maintain the occupancy time for each write port, which is represented as *T*. The overall occupancy time for the processor is represented by Equation (3).

(3) $$\left\{T_{E1},\;T_{E2},\ldots ,T_{EN}\right\}$$

When an instruction is to be issued, the processor could determine its occupancy time for each write port based on the current time with unused write ports having an occupancy time of zero, as represented by Equation (4).

(4) $$\left\{T_{e1},\;T_{e2},\ldots ,T_{eN}\right\}$$

The condition for hardware hazard validation is shown in Equation (5).

(5) $$\sum_{i=1}^{N}\left\{T_{ei}\;\cap \;T_{Ei}\right)\;=0\;$$

### 3.2. Design Proposal

**Figure 6:**
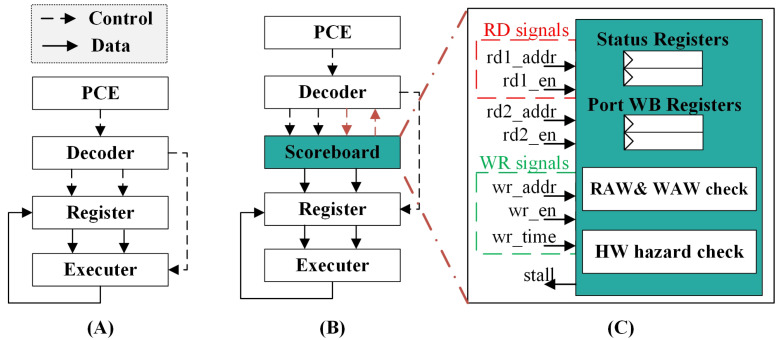
The proposed scoreboard architecture. (**A**) shows the architecture of the original processor, while (**B**) shows the processor architecture with proposed design. (**C**) is the micro-architecture of the scoreboard.

Based on algorithm analysis, we proposed a scoreboard module to address data hazards. Its architecture diagram is shown in Figure 6. This proposal utilizes a centralized scoreboard to predict processor behavior and control instruction dispatch. Figure 6A illustrates the architecture of the original processor, while Figure 6B depicts the architecture after the implementation of this proposal. The architecture incorporates a scoreboard module, which provides a unified instruction issue controller during the instruction dispatch stage.

The scoreboard introduces two new sets of registers: status registers storing the relative write times for corresponding registers and port write-back (WB) registers utilizing a bitmap to track all relative write times for each write port. Additionally, two components are incorporated: a RAW and WAW check for RAW and WAW handling and a hardware (HW) hazard check for detecting simultaneous write-back issues on the same write port, as shown in Figure 6C.

For the scoreboard, each read and write port requires an enable signal and address signal. Furthermore, the write port necessitates a write time (wr_time) signal to determine if the instruction’s write-back conditions are satisfied, simultaneously updating the status register.

Figure 6 only depicts the logic for two register read ports and one register write port. However, the actual circuit requires a complete set for each read and write port.

#### 3.2.1. RAW and WAW Hazard

**Figure 7:**
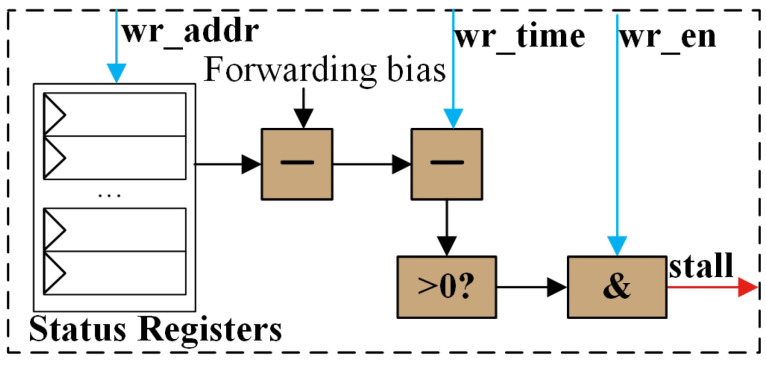
Micro-architecture for each port in RAW and WAW check module.

The logic for the RAW and WAW check is designed to assess whether the current instruction’s read and write actions will cause data hazards. According to the proposed algorithm, this design utilizes the same hardware to evaluate both RAW and WAW conditions. Figure 7 illustrates the micro-architecture for RAW and WAW detection at each port. The distinction between RAW and WAW detection arises from the potential existence of forwarding logic at the read ports. Consequently, RAW detection requires the status value to be adjusted by subtracting the forwarding bias before comparing it with the wr_time to ascertain whether a RAW or WAW hazard exists at the port. In cases of WAW, or when RAW occurs without forwarding logic, the value of the forwarding bias is set to zero.

**Figure 8:**
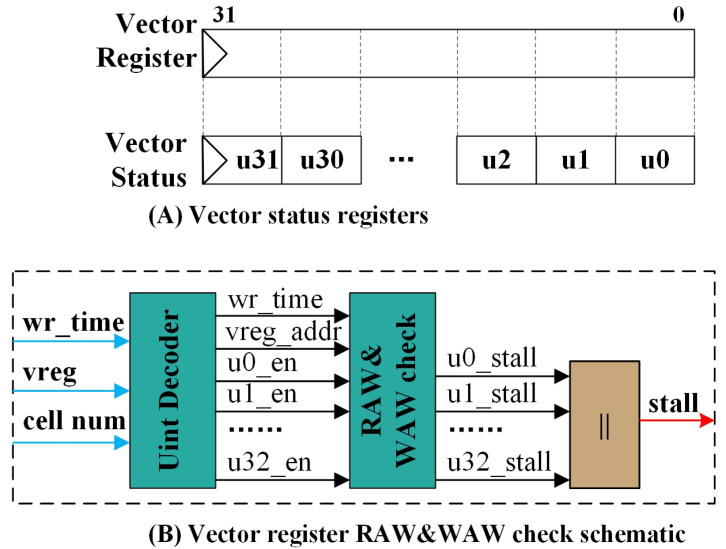
(**A**) Vector register and its status files. (**B**) Schematic for vector registers RAW and WAW check module.

For vector registers, the complexity of register read and write access is notable, as the number of accessible units at the read and write ports is quite flexible. The proposed architecture can be employed to ensure data validity in vector register files. Figure 8A represents the status registers used in the vector register array, which also adopts a vector architecture. Each vector contains 32 units, corresponding to the write-back status of each unit in the vector register.

The schematic for vector register RAW and WAW validation is illustrated by Figure 8B. For the vector register file, the representation of vectors includes a starting address (vreg) and a length (num). The Unit Decoder is required to break down the input starting point and length into individual unit enable signals and vector register addresses. Each unit’s validity must be assessed with arbitration conducted for every unit in each vector read and write operation. The pseudo-code for detecting RAW and WAW hazards in vector registers is presented as Algorithm 1, using a unified representation.   
**Algorithm 1:** Vector register RAW and WAW detection for each port.
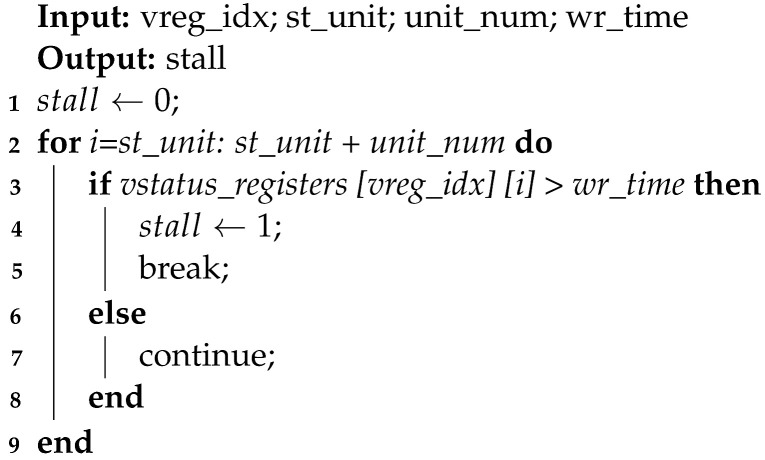


**Figure 9:**
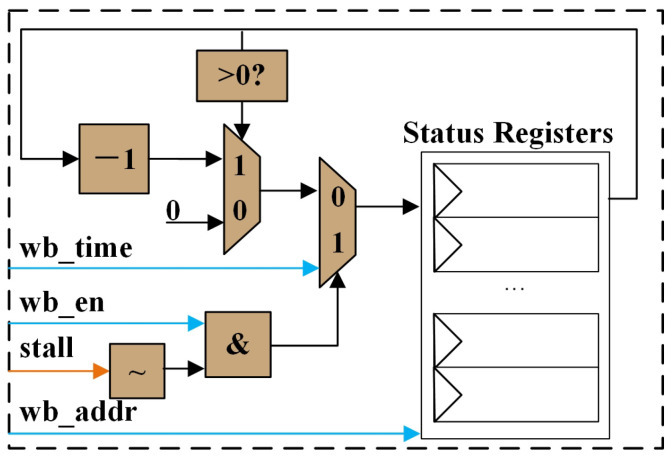
Status register write-back module micro-architecture.

While evaluating WAW, it is necessary to update the corresponding status registers. Figure 9 illustrates the logic for updating the status register for each write port.

When a new write operation occurs, if the final determination from the scoreboard indicates that no stall happens, the value will be updated in the corresponding status register associated with that register. Conversely, if a stall occurs and the status register value is not zero, it will decrement by one; otherwise, the value will remain zero.

To gain a more intuitive understanding of the workflow of this project, Table 1. provides a detailed outline of the various values for the status register of vector register 3, as the processor running in the AS architecture is depicted in Figure 5C.

#### 3.2.2. Hardware Hazard

**Figure 10:**
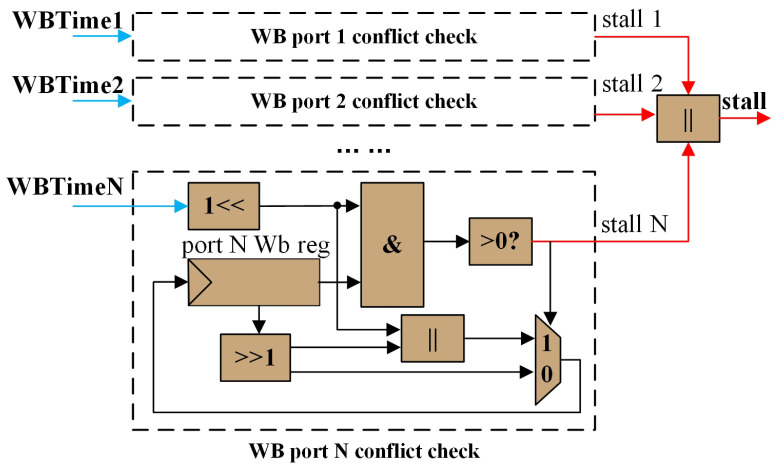
Write-back port write-back conflict detection module micro-architecture.

To address the issue of simultaneous write-backs, this design allows different write ports to write back simultaneously provided they do not target the same register. The RAW and WAW check module ensures that there are no conflicts in writing back to the same register. However, it cannot detect the situation where the same write port attempts to write back to different registers simultaneously. In the scoreboard, we introduced the HW hazard check module to monitor the port write-back status whose micro-architecture is illustrated in Figure 10.

This module uses a bitmap to record the write-back times of the port. Whenever a new write operation is initiated, the module generates a bitmap value representing the current WB time, which is then compared with the port WB register to determine whether there is a conflict in WB timings. If a conflict exists, a stall signal is generated, and the bitmap value in the port WB register is right-shifted by one bit. If no conflict is detected, the bitmap value in the port WB register is also right-shifted by one bit, and the new WB time is updated accordingly. Similar to the status register, the port WB registers for vector registers needs to record the status for each unit of every vector register.

**Figure 11:**
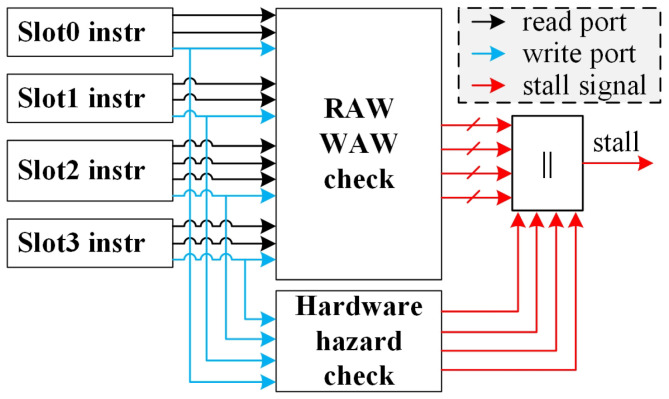
Overall input and output signals for scoreboard.

Figure 11 illustrates the overall input–output interface of this project. For VLIW instruction with several slots, the scoreboard module must perform a RAW check for each read port and a WAW check and a hardware hazard for each write port across all slots. It then integrates all these assessments to determine whether the current VLIW instruction can be dispatched.

## 4. Implementation and Evaluation

**Figure 12:**
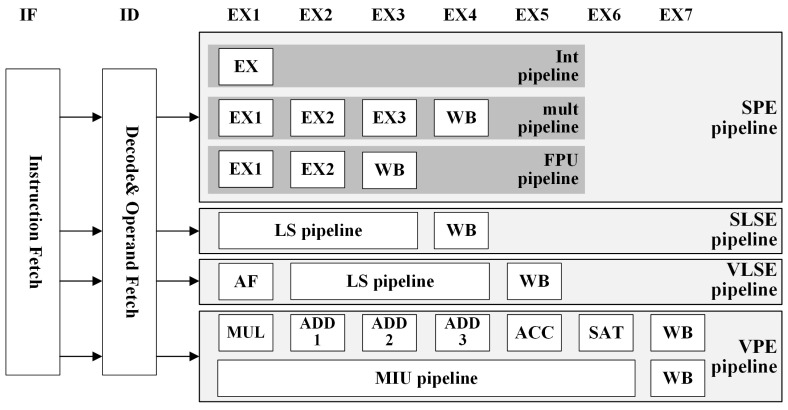
Pipeline implemented in this project.

The Sayram processor is implemented using Verilog. The pipeline design of the Sayram processor discussed in this paper is shown in Figure 12. The load instruction of the scalar takes a total of four cycles to write back to the register, while the vector load requires five cycles. In the original design of Sayram, to facilitate programming, the load instructions for SLSE and VLSE were designed to stall the pipeline before data retrieval. In contrast, the SPE and VPE units are designed for non-blocking pipeline execution, requiring programmers to manually arrange instructions to optimize performance.

To evaluate the execution results, we modified the QEMU simulator [32]. QEMU, developed by Bellard, is a general-purpose processor simulator that accelerates simulation through TCG technology and provides bit-accurate simulation. We added the VPE and VLSE elements to QEMU. For the precise analysis of execution time, we implemented clock-accurate simulation using coroutines [33] in QEMU and designed different pipeline strategies for long pipeline instructions, which are categorized as FS, SWS, and AS.

In the SWS scenario, the absence of hardware data hazard protection can lead to program errors. The order of instructions significantly affects the results of FS and AS. To ensure fairness in experimentation and correct program execution, we developed a scheduler based on the list scheduling algorithm [17]. This tool rearranges instructions according to their dependencies, ensuring that the resulting instruction sequence runs correctly with as few invalid instructions or stalls as possible. The scheduler’s optimization ensures that the instruction sequences in each scenario are the most efficient.

**Figure 13:**
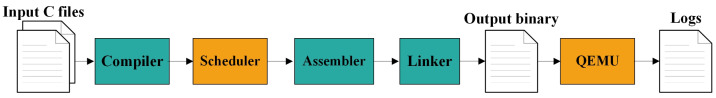
Simulation process.

Figure 13 illustrates the performance evaluation process of this design. To ensure consistency in experimental inputs, we integrated a scheduler component into the compilation process, which is responsible for rearranging the instructions. We then use clock-accurate QEMU for unified simulation, and the execution logs and profiling information will be printed to a log file.

### 4.1. Performance

We conduct three main comparisons: standard C library analysis focusing primary scalar instructions, kernel function analysis emphasizing vector instructions and real application case analysis combining both scalar and vector instructions to reflect the performance of this scheme in real-world scenarios. In addition, we summarize the hazard handling capacities of different designs and compare their performance with competing works.

We performed a comparison of execution time and instruction code size for each experimental group. The execution time of the same program can effectively reflect the processor’s IPC and indicate the performance of the processor under different pipeline strategies. Since DSPs typically employ a Harvard architecture, the instruction code size can reflect the size of the on-chip program memory.

#### 4.1.1. Standard Function

SPECINT and SPECFLOAT are common benchmarks for evaluating general-purpose processors [34]. However, they are not suitable for DSPs that focus on digital signal computation. To assess the performance improvement of this scheme for scalar instructions, we evaluated several functions from the musl standard library [35], including atoi (string to integer), memcpy, and memset, along with four cryptographic programs: Blowfish, MD5, SHA-256, and SHA-512. These standard library functions were implemented in standard C without vector instructions. We compiled using the compiler’s O2 optimization and then rearranged the instructions with the scheduler and analyzed the results using QEMU.

**Figure 14:**
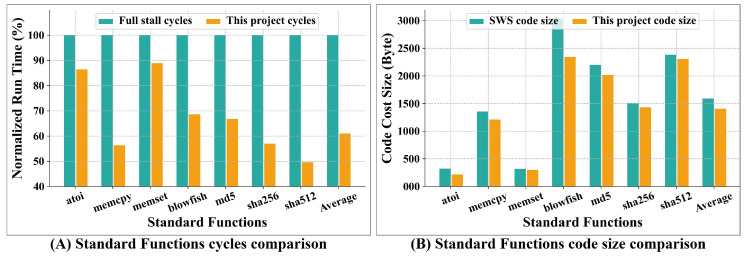
Comparison result for standard functions. (**A**) shows normalized run time comparison between FS and this project, while (**B**) presents code cost size comparison between SWS and this project.

Figure 14A compares the execution times of standard functions in this project with the full stall scenario. Due to the varying execution times of the functions, the displayed values are normalized. Overall, this design achieves an average performance improvement of 38.95%.

The code size can be obtained from the .text section of the compiled ELF executable file. Our compiler scheduler is used in the case of Software Scheduling (SWS) for instruction scheduling. NOP instructions are inserted if two instructions exhibit data hazards and there are no unrelated instructions available for filling the gap. The elimination of NOP insertion is the contributing factor to reduce the program memory cost for AS conditions. Figure 14B compares the instruction memory requirements of this project with those of SWS for standard functions. The average code cost size in this project has been reduced by 11.62%. For DSPs, which often employ Harvard architecture, the program memory is typically SRAM (Static Random Access Memory). The cost size reduction allows the processor to utilize smaller SRAM or enables the same-sized SRAM to accommodate a greater number of instructions.

#### 4.1.2. Kernels

DSPs are primarily utilized for rapid data signal processing for specific domain, and Sayram is a DSP designed for wireless communication signal processing. Let us analyze its performance through wireless communication kernels. Matrix inversion is a common algorithm in the field of wireless communication, which is frequently employed in channel estimation and symbol detection algorithms [36]. Common methods include QR decomposition and LU decomposition. The primary QR decomposition algorithms encompass Givens (GS) and Schmidt (SMT). Kernel group 1 comprises the following kernels: GS2×2, GS2×4, GS4×4, HH2×2, HH2×4, HH4×4, SMT2×2, SMT2×4, SMT4×4, LU2×2, LU3×3, and LU4×4. These operations are performed on one matrix. Kernel group 1 includes the determinants (DET) of 2×2, 3×3, and 4×4 matrices [37]. These test cases predominantly utilize vector instructions and are implemented using handwritten assembly.

**Figure 15:**
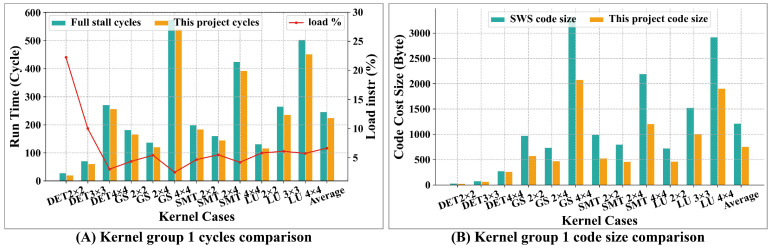
Comparison result for kernel group 1. (**A**) shows normalized run time comparison between FS and this project, while (**B**) presents code cost size comparison between SWS and this project.

Figure 15A presents a comparison of the execution times for kernel group 1 between this project (AS) and the FS scenario with AS achieving an average performance improvement of 8.62%.

Since these kernels are focusing on data computation and the number of load instructions is relatively low, as shown in Table 2, the performance enhancement is not very pronounced. It can also be observed from Figure 15A that as the percentage of load instructions increases, the performance improvement in the AS scenario becomes more evident.

Figure 15B illustrates the comparison of the code size for kernel group 1 between this project and the SWS scenario where software scheduling is employed. This project achieves a 38.04% reduction in code cost.

Matrix multiplication is also a widely used operation in scientific computing. We evaluated the kernels for matrix multiplication with dimensions including 4 × 4, 5 × 5, 6 × 6, 7 × 7, 8 × 8, 9 × 9, 10 × 10, 11 × 11, and 12 × 12, each involving a single matrix multiplication (kernel group 2). The process for these kernels involves loading data from memory, performing mathematical operations, and then writing the results back to memory.

**Figure 16:**
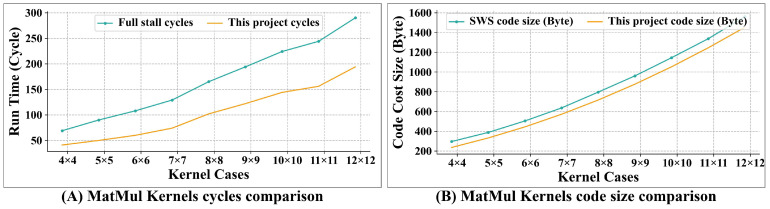
Comparison result for matrix multiplication kernels. (**A**) shows normalized run time comparison between FS and this project, while (**B**) presents code cost size comparison between SWS and this project.

Figure 16A presents a comparison of the execution times for matrix multiplication kernels between this project and the FS scenario with this project achieving an average performance improvement of 37.67%.

Figure 16B illustrates the comparison of the code size for matrix multiplication kernels between this project and the SWS scenario, where software scheduling is employed. This project achieves a reduction in code cost by 9.13%.

**Figure 17:**
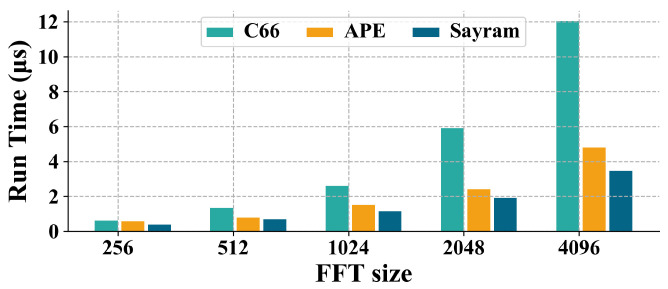
The 16-bit complex fixed point FFT performance.

We have also simulated various typical signal processing algorithms on Sayram and compared its performance with that of the commercial TI C66x core [9] and APE [38], which is a DSP featuring similar process nodes and computational resources. Sayram and APE both operate at 1 GHz, while the C66x core runs at 1.25 GHz. The performance data for the C66x and APE were obtained from ref. [38]. Figure 17 presents the execution time of a complex 16-bit fixed-point FFT algorithm. Using the scoreboard proposed in this paper, the Sayram processor achieves an average speedup of 2.97× (times). compared to the C66 core and 1.33× compared to APE for fixed-point FFT.

#### 4.1.3. Real Application

The following simulation utilizes real 5G application programs, analyzing from both runtime and code cost perspectives. These benchmarks include the 5G mobile communication base station’s symbol processing programs: Channel State Information Reference Signal (CSIRS), Demodulation Reference Signal (DMRS), Physical Broadcast Channel (PBCH), Physical Downlink Control Channel (PDCCH), Physical Downlink Shared Channel (PDSCH), Physical Random Access Channel (PRACH), Physical Uplink Control Channel (PUCCH), Physical Uplink Shared Channel (PUSCH), Precode, and Sounding Reference Signal (SRS) [16]. Due to the differences in architecture and instructions set among DSPs, and the common use of built-in functions for programming in specific domains, each vendor develops its own applications. So, we manually developed these programs based on the 3GPP standards [16]. These applications are programmed using built-in functions, with core computations accelerated through vector instructions, allowing for a more effective assessment of the overall performance improvement achieved with this solution.

**Figure 18:**
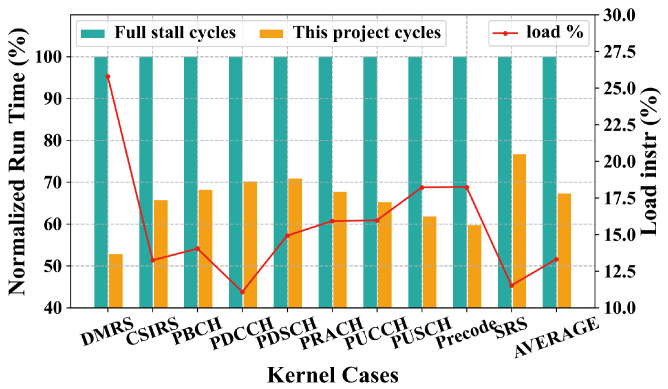
Normalized run time comparison between FS and this project for real application.

Due to the varying execution times of the functions, the displayed values are normalized. From the experimental results, it can be observed from Figure 18 that after implementing this solution, the average runtime decreased by 32.75% compared to the full stall scenario. Notably, the DMRS, which has high memory access requirements as the red lines showed in Figure 18, saw a runtime reduction of 47.21%.

Table 3 presents a comparison of code cost for real applications between this project and the SWS scenario. From the perspective of common 5G baseband algorithms, on average, this project has achieved a size reduction of 19.56%.

#### 4.1.4. Hazard Handling Capability

The following summarizes the hazard handling capabilities, as shown in the Table 4. In summary, the proposed approach enables global hazard handling that is not limited to consecutive instructions; it eliminates the need for software intervention for hazard handling. Our solution encompasses RAW, WAW, and hardware hazard detection. Additionally, we provide a fine-grained detection method for reading and writing vector register files, enhancing the overall efficiency of processor operation.

### 4.2. Synthesis Result

This design utilizes the TSMC 12 nm process library for synthesis. Based on the original processor design, additional logic has been integrated as outlined in this paper. The table presents the area of the scalar register files, vector register files and the whole processor, as well as the area of the scoreboard for both the scalar and vector portions, along with the total scoreboard overhead. The detailed data are shown in Table 5. This processor includes a register file with 32 registers, each 32 bits wide, a vector register file with 32 registers, each 512 bits wide, 128 KB of program memory (PM), and 512 KB of data memory (DM). The whole processor area comprises the areas of the PM, DM, and the peripheral bus.

Sayram employs 128 K of instruction memory, which measures 116,383 μm^2^, representing 9.8% of the total processor area. Compared to the SWS approach, this design achieves a 19.56% reduction in program memory, corresponding to a savings of 1.92% in total area. Thus, relative to the SWS method, this design reduces silicon overhead by 1.92%−0.91%=1.01%.

From the data in Table 5, it is evident that the scalar scoreboard accounts for 18.4% of the scalar register area, while the vector scoreboard constitutes approximately 16.2% of the vector register file. The design occupies 0.91% of the total processor area.

With the inclusion of the scoreboard, the performance improvement is 32.75% as evaluated in Section 4.1.3. The performance-to-cost trade-off factor can be evaluated as (1+32.75%)/(1+0.91%)=132%.

With the addition of the scoreboard proposed in this paper, the area of Sayram increased slightly. Table 6 presents the final synthesis results for Sayram alongside the results for APE [38]. From the table, it is evident that Sayram has a significant power efficiency advantage over APE with a 62.96% reduction in normalized area compared to APE.

## 5. Conclusions

DSPs are increasingly embraced in areas such as artificial intelligence, communications, media, gaming, and control systems. Instruction parallel (VLIW) and data parallel (SIMD or vector) DSPs achieved good performance. To obtain better performance, we developed ASIP architectures. With its deep pipeline, we further reached significant performance through instruction fusion. However, the ASIP datapath with a deep pipeline introduces challenges, which we categorized into three main areas: handling RAW hazards, managing hardware hazards, and addressing WAW hazards. Solutions can be implemented using advanced compiler techniques and hardware hazard management. In this paper, we introduced both solutions and proposed a solution to address these challenges.

The proposed design utilizes a single scoreboard to simultaneously address both RAW and WAW issues. This scoreboard is applicable to scalar and vector registers in specialized processors. Its lightweight characteristics meet the low-power and low silicon consumption demands of processors. This paper proposes a scoreboard design to avoid data hazards in long-pipeline processors with multiple read and write ports, making it suitable for ASIP SIMD and vector architectures. This architecture enhances processor performance with minimal silicon overhead and design complexity. The module significantly improves processor efficiency with the performance-to-cost trade-off factor is estimated at 132%.

## Figures and Tables

**Table 1:** Status value varies with time for vector register 3.

Time	vreg3.0-31 Status Value	Action
**t1**	0	Init value.
**t2**	5	Write pipeline in to status reg
**t3**	4	Status minus 1. There is no potential hazard for the second instruction so it could be issued.
**t4**	3	Status minus 1. Input pipeline value is 0, which is less than status value. So the output stall value is 1.
**t5**	2	Same as above.
**t6**	1	Same as above.
**t7**	0	Input pipeline value is 0, which is equal to status value of unit status. So the last could be issued.

**Table 2:** The proportion of load instructions used in the case studies.

**Kernel**	**DET2 × 2**	**DET3 × 3**	**DET4 × 4**	**GS2 × 2**	**GS2 × 4**	**GS4 × 4**				
**Load %**	22.22%	10.00%	3.01%	4.35%	5.41%	2.48%				
**Kernel**	**SMT2 × 2**	**SMT2 × 4**	**SMT4 × 4**	**LU2 × 2**	**LU3 × 3**	**LU4 × 4**				
**Load %**	4.65%	5.48%	4.19%	5.80%	6.08%	5.73%				
**Kernel**	**CSIRS**	**DMRS**	**PBCH**	**PDCCH**	**PDSCH**	**PRACH**	**PUCCH**	**PUSCH**	**Precode**	**SRS**
**Load %**	13.24%	25.79%	14.03%	11.08%	14.92%	15.91%	15.96%	18.21%	18.23%	11.52%

**Table 3:** Code cost size comparison between SWS and this project for real application.

Case	SWS Code Size (Byte)	This Project Code Size (Byte)	Reduction
DMRS	7728	6612	14.44%
CSIRS	1400	1240	11.43%
PBCH	3260	2708	16.93%
PDCCH	4320	3824	11.48%
PDSCH	15,184	12,132	20.10%
PRACH	5524	4400	20.35%
PUCCH	30,832	25,988	15.71%
PUSCH	14,648	11,744	19.83%
Precode	4492	3872	13.80%
SRS	36,664	27,264	25.64%
Average	12,405	9978	19.56%

**Table 4:** Hazard handling capabilities.

Related Works	Hardware Capabilities	Need to Rely on Software
TI C66 [9]	RAW detection between consecutive instructions	Yes
Hexagon HVX [28]	RAW detection between consecutive instructions	Yes
Ref. [20]	RAW and WAW detection for short pipeline instructions	No
SiMT-DSP [21]	RAW detection between consecutive instructions	Not mentioned
Ref. [22]	Coarse-grained RAW and WAW detection for vector register	No
Proposed work	RAW, WAW, HW hazard globally detection regardless of the instruction length	No

**Table 5:** Synthesis result comparison with original design.

	Original Area (μm^2^)	This Project Increased Area (μm^2^)	This Project Increased Area Percentage
Scalar register file	7960	1465	18.40%
Vector register file	57,512	9317	16.20%
Program memory (128 KB)	116,383	-	-
Data memory (512 KB)	503,386	-	-
Whole processor	1,183,734	10,782	0.91%

**Table 6:** Synthesis result comparison with other studies.

Research	Frequency (GHz)	Process (nm)	Area (mm^2^)	Normalized Area (mm^2^) ^1^	Power (mW)
APE [38]	1	40	36	36	0.7
Our work	1	12	1.2	13.3	0.098

^1^ $Normalized\_\;area\;=area\;\times {\left(\frac{40\;nm}{process}\right)}^{2}$.

## Data Availability

The data that support the findings of this study are available on request from the corresponding author.
